# Confidence judgments interfere with perceptual decision making

**DOI:** 10.1038/s41598-024-64575-7

**Published:** 2024-06-19

**Authors:** Kit S. Double, Damian P. Birney

**Affiliations:** School of Psychology, University of Sydney, Sydney, Australia

**Keywords:** Confidence, Confidence judgments, Metacognition, Reactivity, Perceptual discrimination, Psychology, Human behaviour

## Abstract

Determining one’s confidence in a decision is a vital part of decision-making. Traditionally, psychological experiments have assessed a person’s confidence by eliciting confidence judgments. The notion that such judgments can be elicited without impacting the accuracy of the decision has recently been challenged by several studies which have shown reactivity effects—either an increase or decrease in decision accuracy when confidence judgments are elicited. Evidence for the direction of reactivity effects has, however, been decidedly mixed. Here, we report three studies designed to specifically make reactivity effects more prominent by eliciting confidence judgment contemporaneously with perceptual decisions. We show that confidence judgments elicited contemporaneously produce an impairment in decision accuracy, this suggests that confidence judgments may rely on a partially distinct set of cues/evidence than the primary perceptual decision and, additionally, challenges the continued use of confidence ratings as an unobtrusive measure of metacognition.

## Introduction

A key component of human cognition is our ability to judge the confidence we have in our decisions^1^. Confidence in our decisions closely tracks objective accuracy even in the absence of feedback^2,3^. Confidence is viewed as an internal signal of the probability that a decision was correct—one that is assumed to be computed automatically and effortlessly when a decision is made^4^. For over a century, researchers interested in subjective confidence have elicited confidence judgments as means to assess participants’ performance monitoring and subjective sense of accuracy^5^. Confidence judgments continue to provide vital insights in a range of domains including, metacognition, memory, reasoning, learning, and perception^6^. However, recently researchers have begun to ask whether eliciting confidence ratings might unintentionally affect participants’ decisions; if it does, this is known as *reactivity*. Evidence exists both in support^7–14^ and against^15,16^ the idea that confidence judgments are reactive. Reactivity effects have also been observed in other ratings used to measure subjective performance^17–21^. Reactivity poses a significant challenge for the vast number of researchers across many fields interested in subjective confidence.

Confidence judgments explicitly require participants to monitor their confidence. Many theories of decision-making have argued that the computation of confidence happens automatically^4^, or even that the accumulation of confidence can better explain decision-making than the accumulation of actual decision evidence^22^. This view is challenged by consistent evidence that eliciting retrospective confidence ratings increases decision response time^9–14,23^. For example, in a binary decision task, Baranski et al. found that eliciting retrospective confidence ratings increased decision time by 23–24%^13^. These findings have been interpreted as suggesting that monitoring confidence adds an additional computational burden, specifically the need to monitor one’s performance in order to make an accurate confidence judgment, which competes for cognitive resources that are needed to make the primary decision. Reactivity, therefore, suggests that the evidence needed to judge one’s confidence is distinguishable, at least in part, from the evidence needed to make the primary decision^13,23^.

What remains less clear is whether judging one’s confidence ultimately facilitates or impairs the accuracy of the primary decision. Several researchers have argued that the overlap between the information used to judge one’s confidence and the information used to make the primary decision determines the direction of reactivity effects on accuracy^17,18,24^. If eliciting confidence ratings slows decision-making but facilitates accuracy it suggests that the information used to judge one’s confidence *complements* the information used to make the primary decision. On the other hand, if eliciting confidence ratings slows decision-making and impairs accuracy it suggests that the information used to judge one’s confidence *competes* with the information needed to make the primary decision. Alternatively, there may be no effect of eliciting confidence ratings on accuracy, which would support the currently accepted view that confidence and decision-making are based on the same shared process^22^.

Unfortunately, the current evidence is equivocal with regards to the effect of confidence ratings on accuracy, with some studies finding that eliciting confidence ratings produces small reductions in accuracy, others have found small increases in accuracy, while still others have found no effect on decision accuracy^7–9,11,13,15,16,23^. This may be due to contextual factors that influence what evidence is used to judge one’s confidence. For example, Double & Birney showed that the effect of confidence ratings on accuracy depends on how the confidence judgment is worded^8^. Yet the inconsistency of these effects limits what reactivity effects can reveal about the basis on which individuals judge their confidence and how confidence fits within models of decision-making. It has also led to some doubting whether reactivity effects on accuracy are replicable^25^.

Reactivity to confidence ratings has thus far been shown in *retrospective* confidence ratings (ratings made after the primary decision). This is important to consider when judging the reliability and robustness of reactivity effects because the size of these effects is likely to be muted by the fact that, when confidence ratings are elicited retrospectively, reactivity can only be plausibly driven by the *repeated* requirement to judge one’s confidence in-between trials. This is to say, reactivity as it is currently studied is essentially the reactivity effect of all previous confidence ratings on the decision accuracy of a given trial, whereby the previous requirement to judge one’s confidence changes the accuracy with which participants make *subsequent* decisions (e.g. decision accuracy on trial 5 can only be reactive to the requirement to rate one’s confidence on trials 1–4, as well as the *presumption* that one will need to rate one’s confidence after trial 5 is complete and not the actual act of rating one’s confidence to trial 5, which has not occurred when the decision on trial 5 is made). While the effect of retrospectively elicited confidence ratings is interesting, a more robust estimate of the effect of judging one’s confidence on decision accuracy will be gained if confidence is judged more proximally to the decision. Importantly, reactivity to such judgments will also imply something about the nature of the evidence used to make confidence ratings; positive reactivity suggests that this evidence complements the evidence needed to make the primary decision, while negative reactivity implies a divergence in the evidence used to make the confidence rating when compared to the primary decision.

To produce a clearer picture of the effect of judging one’s confidence on decision-making the current studies utilize both retrospective confidence judgments (RCJ) as well as contemporaneous confidence judgments (CCJ), whereby the decision and confidence judgment rating are made concurrently. CCJ have been used previously in the study of decision confidence^26–29^. For example, in a binary location decision-making task participants might select from 4 options: “1 = Definitely Left”, “2 = Maybe Left”, “3 = Maybe Right”, “4 = Definitely Right”. Compared to RCJ, CCJs should provide a much more robust picture of the effect of judging one’s confidence on decision accuracy and allow for comparison of reactivity under RCJ versus CCJ. Specifically, if eliciting confidence judgments produces better or worse decision-making then it should do so much more prominently when participants need to produce both a decision and a confidence judgment concurrently. Importantly, in the current paradigm decision time is unlimited, thus any reactivity to CCJs we observe is not simply due to the additional time required to judge one’s confidence as opposed to simply making a binary decision (difference in response time could simply be explained by the complexity of the motor response required when making a CCJ, if nothing else). Indeed, the central research question addressed by the current study is whether reliable and robust reactivity effects occur when participants are asked to judge their confidence contemporaneously when making a perceptual decision.

## Experiment 1

### Method

#### Participants

Undergraduate psychology students from an Australian university participated in the study in exchange for course credit. A power analysis using G*Power (within-person t-test; 80% power, effect size d = 0.50) was performed to determine the sample size. Given that no previous study has examined reactivity effects using concurrent confidence ratings, we based our power analysis on the assumption that effects would be somewhat larger than those observed for retrospective confidence ratings^21^. The power analysis indicated that a total sample size of 34 was required. In order to account for potential non-serious responses a total sample of 40 was recruited (M_age_ = 21.4, SD = 6.0, 78% female).

### Materials

**Figure 1:**
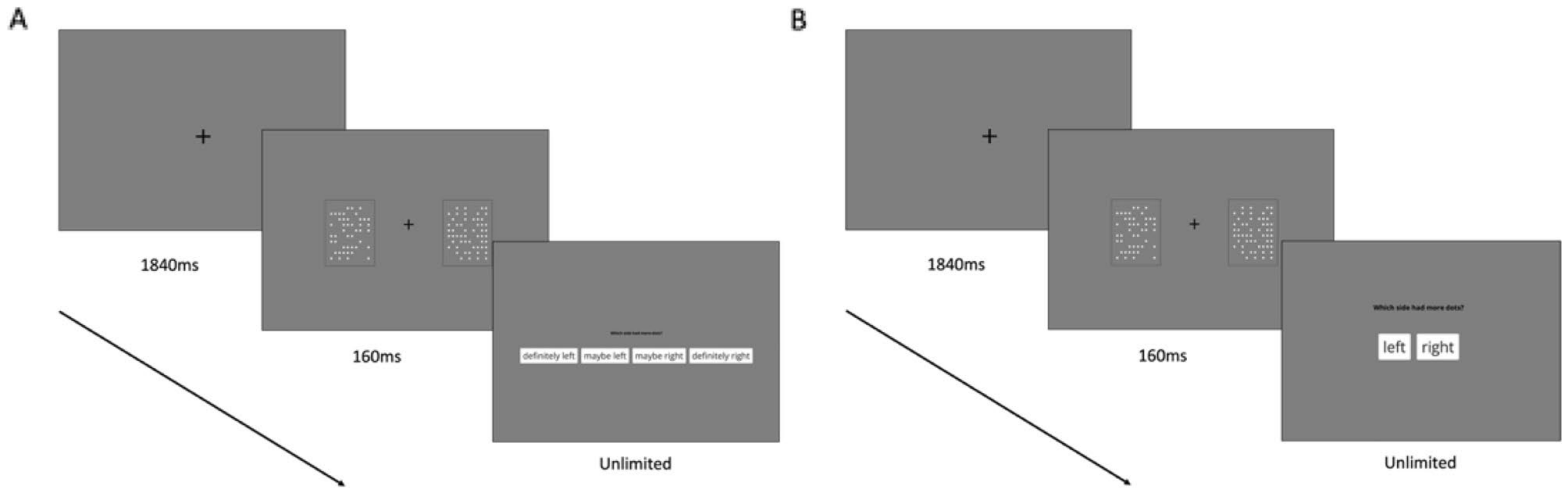
Experimental sequence for the (**A**) contemporaneous confidence ratings condition and (**B**) control condition in Experiment 1.

The experimental task was a two-alternative forced-choice dot discrimination task. In the task, participants were briefly presented with two squares each containing a display of white on a gray background, see Fig. 1. Participants were tasked with deciding whether there were more dots in the square on the left or the right side. Each trial began with a fixation cross in the center of the screen for 1840 ms before the two squares were presented for 160 ms. The configuration of the dots in each of the squares was randomly determined for each trial. The configuration was programmed to present the dots with a 10 × 10 grid of potential positions. Each dot was displayed in size 60 pt ‘Open Sans’ font. Each trial had a fixed dot difference of ten dots, with either the left or right square randomly determined to have ten more dots than the other (number of dots were 45 and 55 for each square). Participants were able to respond immediately after stimulus offset. The response options depend on the block condition. In control condition blocks, participants were asked ‘Which side had more dots?’ with two buttons ‘left’ and right’ presented on screen. In the confidence rating blocks, participants were asked the same question, but four buttons were presented: 'definitely left', 'maybe left', 'maybe right', 'definitely right'. Participants made their selection with the mouse in both conditions. There was no time limit for participants to make their responses. A blank screen was then displayed for 1500 ms before the next trial commenced. Accuracy was computed by disregarding the degrees of confidence in the response (e.g., 'definitely left' and 'maybe left' were both treated as a ‘left’ response).

#### Procedure

All research was performed in accordance with the Declaration of Helsinki. The study was approved by the Human Research Ethics Committee at The University of Sydney. Informed consent was obtained from all participants. Participants completed the study on their personal computers. Stimuli were coded using jsPsych and programmed to display in a standardized fashion. Participants first provided basic demographic variables (age and sex) before receiving instructions about the task. Participants then completed a practice block of 12 items with feedback in order to familiarize themselves with the task. Participants then completed six blocks of 36 trials each. As described above, three blocks required participants to make only the perceptual decision, while the other three blocks required participants to make the perceptual choice concurrently with the confidence judgment. The order of conditions was counterbalanced across participants such that they were randomly allocated to begin with either a control or CCJ block and then completed the other blocks in an alternating fashion (e.g., CCJ-Control–CCJ-Control).

#### Openness and transparency

For all experiments, we have reported all measures, conditions, data exclusions, and how we determined our sample sizes. Data and materials are available on the Open Science Framework https://osf.io/3s98e). These experiments were not preregistered.

### Results and discussion

One participant performed at chance (50%) and was removed from the analysis. Bayes Factors are computed using ‘bayesFactor’ package in R with the default priors.

Response times were log-transformed prior to analysis. Trials with response times greater than 3 standard deviations from the mean were excluded from the response times analysis (3 responses). When analyzing response time data, we included response accuracy as a factor. A 2 (condition: CCJ vs Control) × 2 (accuracy: correct vs incorrect) within-subjects ANOVA indicated that the CCJ group (M = 1550 ms, SD = 639 ms) were significantly slower to respond than the Control group (M = 1167 ms, SD = 488 ms), F(1,39) = 58.93, η_p_^2^ = 0.602, *p* < 0.001, BF_10_ > 100. Responses were significantly slower for incorrect response compared to correct responses, F(1,39) = 18.10, η_p_^2^ = 0.317, *p* < 0.001, BF_10_ > 100. In addition, the interaction between accuracy and condition was significant such that the negative impact of CCJs on response time was more pronounced for accurate response, albeit with weak evidence in favor of the null, suggesting that the response should not be interpreted due to insufficient data, F(1,39) = 5.06, η_p_^2^ = 0.115, *p* = 0.030, BF_10_ = 0.403.

**Figure 2:**
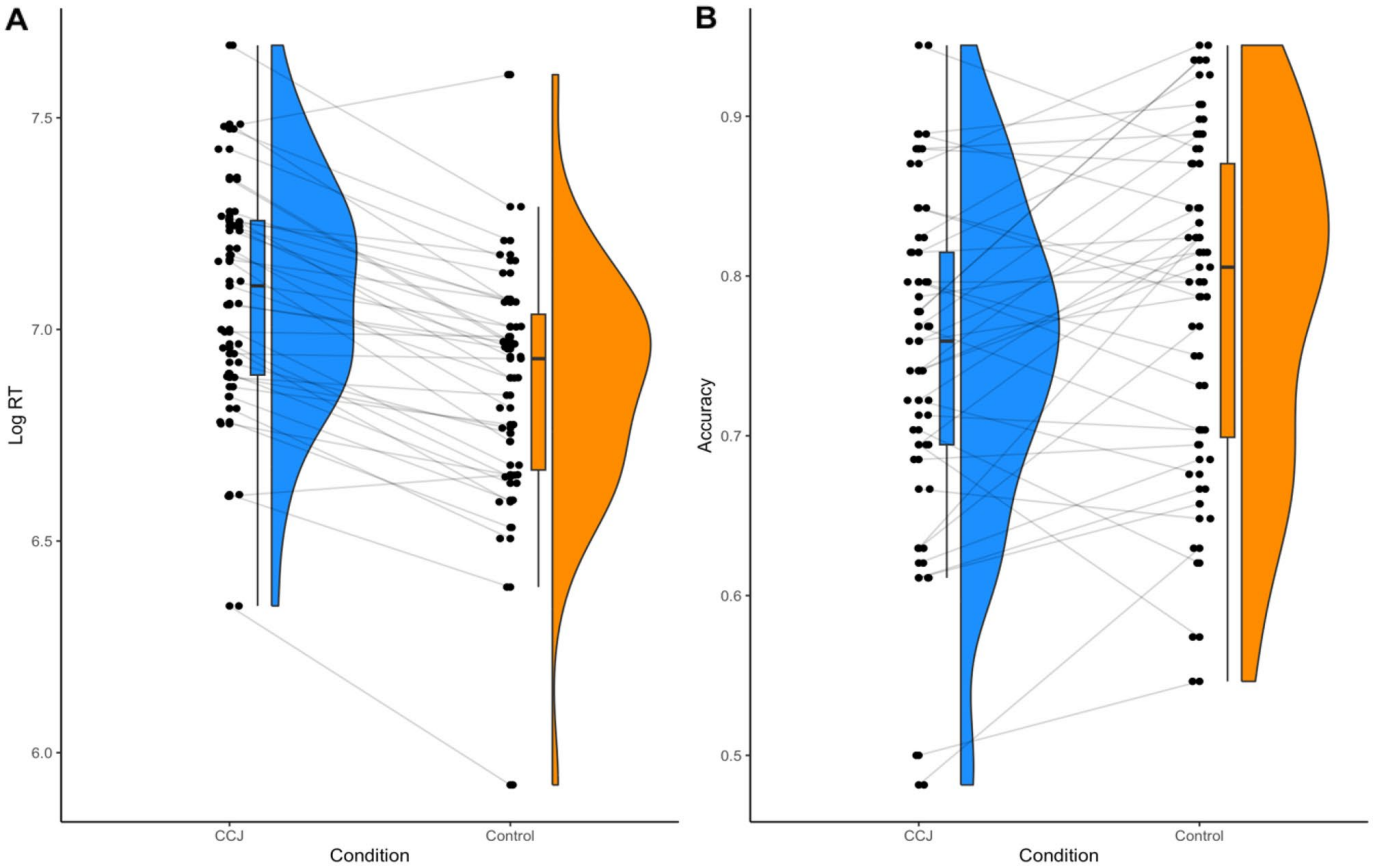
Experiment 1 (**A**) log response times and (**B**) decision accuracy as a function of experimental condition.

Furthermore, responses in the CCJ condition (M = 75%, SD = 10.6) were significantly less accurate than the Control condition (M = 78%, SD = 10.4), t(38) = 2.85, *p* = 0.007, *d* = 0.46 (CI: 0.12 to 0.78), BF_10_ = 5.57, see Fig. 2. These findings suggest that rating one’s confidence produced significantly slower and less accurate decisions. This supports the notion that making concurrent confidence judgments interferes with perceptual decision accuracy.

## Experiment 2

The results of Experiment 1 indicate a clear reactivity effect when CCJ are elicited. While this suggests that making a decision and a confidence judgment simultaneously impairs performance, it is not clear whether rating one’s confidence retrospectively produces similar negative reactivity. As mentioned, the current literature is equivocal about the direction and magnitude of reactivity to retrospective confidence ratings and effects appear to differ based on task and judgment features^8^. While no experiment is going to provide complete certainty about the direction of reactivity, it remains pertinent to see whether reactivity effects are the same direction and magnitude for retrospective and concurrent confidence judgments when the sample and task are the same. As we suggested previously, reactivity effects are likely to be larger when the confidence judgment is elicited at the time of the decision, rather than relying on cumulative retrospective effect produced by the repeated requirement to rate one’s confidence. In Experiment 2, we directly compare RCJs with CCJs, we hypothesize that RCJs will produce a smaller negative reactivity effect than CCJs.

### Method

#### Participants

Recruitment was the same as Experiment 1, with 40 participants (M_age_ = 21.15, SD = 6.03; 80% female) completing the study.

### Materials and procedure

The materials and procedure were the same as Experiment 1 with the following exceptions. An additional RCJ condition was included where participants made the primary decision (using the same binary ‘left’ vs ‘right’ choice as the Control condition) but then made a retrospective confidence judgment. The retrospective confidence judgments were untimed, and participants were asked ‘*How confident are you that your last response is correct?*’. Participants responded with the mouse, selecting between two response options: ‘definitely’ and ‘maybe’. Participants completed six blocks (two of each condition) in the same counterbalanced fashion as Experiment 1.

### Results and discussion

**Figure 3:**
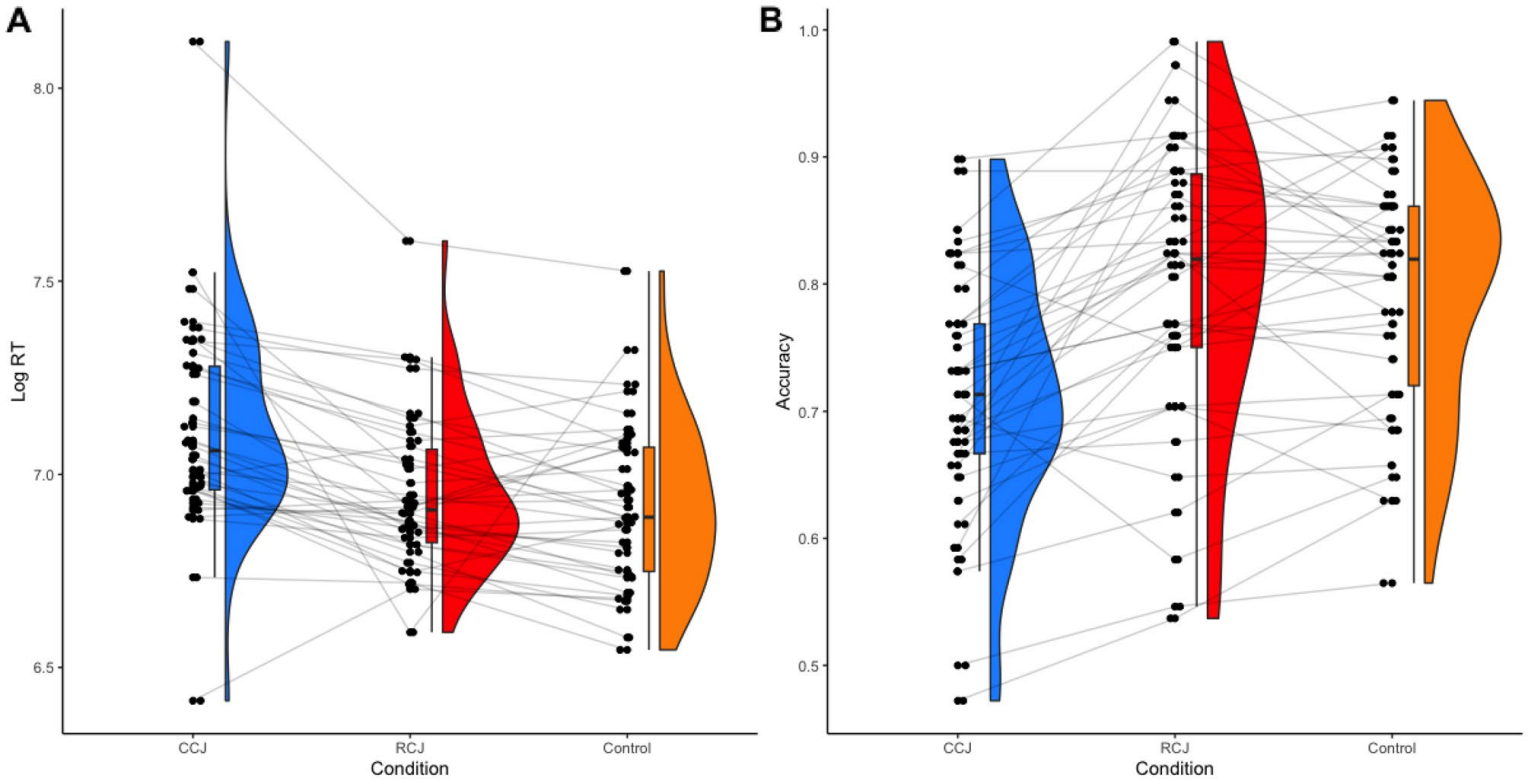
Experiment 2 (**A**) log response times and (**B**) decision accuracy as a function of experimental condition.

Two participants scored at or below chance and were removed from the analysis. Response times were log-transformed prior to analysis. Trials with response times greater than 3 standard deviations from the mean were excluded from the analysis (5 responses). A two-way within-subjects ANOVA 2 (condition: RCJ vs CCJ vs Control) × 2 (accuracy: incorrect vs correct) indicated that there were significant differences in response times between the conditions, F(2,78) = 17.41, *p* < 0.001, η^2^ = 0.309, BF_10_ > 100. Post-hoc pairwise comparisons with Holm correction indicated that indicated that the CCJ condition (M = 1696 ms, SD = 1230) had significantly slower response times than either the Control condition (M = 1413 ms, SD = 841; t = 7.52, *p* < 0.001, BF_10_ > 100) and the RCJ condition (M = 1412 ms, SD = 682; t = 5.24, *p* < 0.001, BF_10_ > 100). The RCJ condition and the control condition did not differ significantly from each other, t = 1.14, *p* = 0.262, BF_10_ = 0.318, see Fig. 3. There was a significant main effect of accuracy, such that responses times were slower for incorrect responses, F(1,39) = 46.94, *p* < 0.001, η^2^ = 0.546, BF_10_ > 100 and the interaction between accuracy and condition was significant, F(2,78) = 3.82, *p* = 0.026, η^2^ = 0.089, BF_10_ = 0.268, with slower responses form the CCJ group again more pronounced for correct responses.

Similarly, a one-way within-subjects ANOVA (condition: RCJ vs CCJ vs Control) indicated that there were significant differences in decision accuracy between the conditions, F(2,74) = 25.98, *p* < 0.001, η^2^ = 0.412, BF_10_ > 100. Post-hoc pairwise comparisons with Holm correction indicated that the CCJ condition (M = 71.3%, SD = 9.8) had significantly lower decision accuracy than either the Control condition (M = 79.2%, SD = 9.3; t = 5.75, *p* < 0.001, BF_10_ > 100) and the RCJ condition (M = 79.8%, SD = 11.3; t = 5.71, *p* < 0.001, BF_10_ > 100). The RCJ condition and the control condition did not differ significantly from each other, t = 0.516, *p* = 0.609, BF_10_ = 0.20. These results again provide strong evidence that CCJs impair decision accuracy. Importantly, we find strong evidence in support of the null that decision accuracy in the RCJ condition does not differ significantly from the Control condition.

These findings suggest that RCJs did little to affect either the response time or the accuracy of the primary perceptual discrimination task. Conversely, the CCJs again slowed responding and impaired decision accuracy. The finding that RCJs do not affect response times is in contrast to previous perceptual discrimination tasks^11,13,23^. However, the current task is distinct from those used previously in that response time was unconstrained by time limits. Furthermore, the current study is the first to examine reactivity in perceptual discrimination using a within-subject design. It may be that the requirement to evaluate confidence on some blocks but not others limited the cumulative retrospective effects of RCJs. Regardless, the findings again confirm that asking participants to judge their confidence at the same time as making the primary decision impairs response accuracy.

## Experiment 3

We have used the data from the previous studies to argue that judging one’s confidence at the same time as making a perceptual decision impairs decision accuracy. However, one potential confound in the previous studies is the complexity of the response options. It may be that having to decide from four response options is simply more difficult than selecting from two options. While we suspect this is unlikely to be true, we designed Experiment 3 to test whether reactivity to CCJs is purely due to the complexity of the response options.

To do this we introduced an additional manipulation whereby some participants were given a very large post-stimulus interval before presenting the response options. Participants in the large post-stimulus interval condition are thus effectively able to make the perceptual decision prior to seeing the response options. If the complexity of the response options is responsible for the reactivity we are observing, then increasing the post-stimulus interval should not matter (there are four response options in the CCJ condition, regardless of post-stimulus interval) and we should continue to observe negative reactivity. However, if it is the *simultaneous* requirement to make a decision and judge one’s confidence that is driving the negative reactivity, then we should not observe reactivity when we increase the post-stimulus interval because the decision is likely to have been made prior to the response options with the confidence judgment being displayed.

We therefore repeated the design of Experiment 1, but this time added an additional between-subjects manipulation. Half of the participants completed the study in the standard fashion, with the immediate presentation of the CCJ after stimulus offset, while the other half completed the task in a delayed condition which had a 4500 ms interval between stimulus offset and the presentation of the CCJ. We hypothesized that if judging one’s confidence at the same time as making a perceptual decision produces negative reactivity rather than the complexity of the response options, then we should observe negative reactivity in the standard condition but not the delayed condition.

### Method

#### Participants

Recruitment was the same as the previous experiments, with 80 participants (M_age_ = 19.86, SD = 2.25; 74% female) completing the study. Participants were randomly assigned to complete the dot discrimination task with either a wait interval between stimulus offset (n = 40) or no wait interval (n = 40).

### Materials and procedure

The materials and procedure were the same as Experiment 1 (i.e., participants completed blocks in either a concurrent confidence ratings or control condition). In addition, a between-subjects manipulation of a wait interval between stimulus offset and presentation of the response options was introduced. In the wait-interval condition, participants saw a blank screen for 4500 ms after stimulus offset before the response options appeared (either ‘Left’ and ‘Right” or “Definitely Left’, ‘Maybe Left’, ‘Maybe Right’, ‘Definitely Right’ depending on block condition). 4500 ms was selected as the wait interval based on piloting because it was long enough to ensure that participants could complete the evidence accumulation process before viewing the response options but was not so long as to unnecessarily burden participants. Participants in the wait-interval condition were therefore encouraged to continue making the perceptual decision before being presented with the (confidence) decision.

### Results and discussion

One participant performed below chance and was excluded from the analysis. A set of 2 (Condition: CCJ vs Control) × 2 (wait vs no-wait) × 2 (accuracy: correct vs incorrect) within-between-within mixed ANOVAs were used to analyze differences in response times.

**Figure 4:**
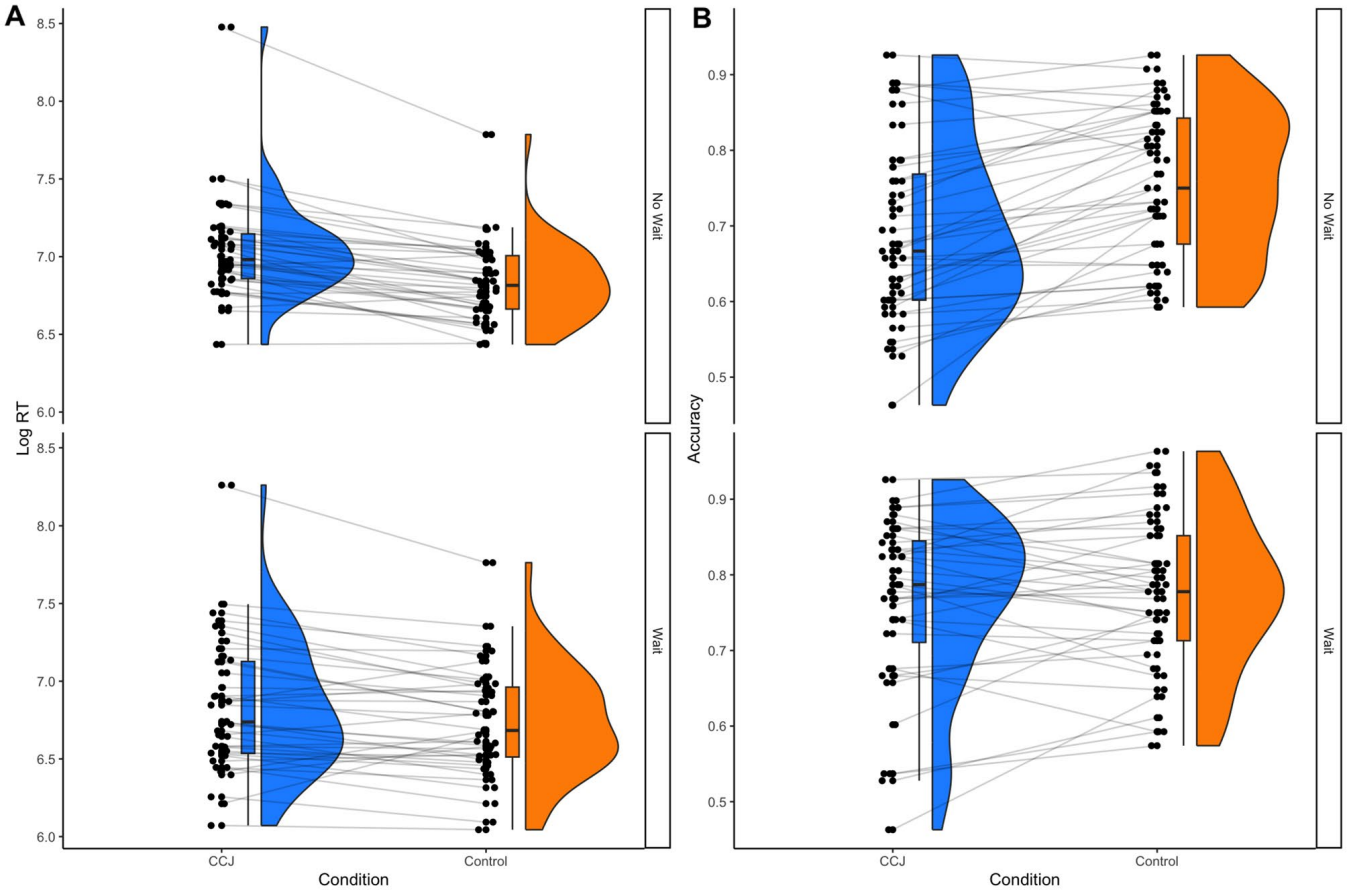
Experiment 3 (**A**) log response times and (**B**) decision accuracy as a function of experimental conditions.

Response times were log-transformed prior to analysis. Trials with response times greater than 3 standard deviations from the mean were excluded from the analysis (3 trials). The mixed ANOVA indicated that when participants were in the CCJ condition (M = 1847 ms, SD = 1789 ms) they were significantly slower to respond than when they were in the Control group (M = 1447 ms, SD = 1290 ms), *F*(1,78) = 36.22, *p* < 0.001, η_p_^2^ = 0.317, BF_10_ > 100. The main effect of wait interval was not significant, *F*(1,78) = 3.59, *p* = 0.062, η_p_2 = 0.044, BF_10_ = 1.28. The main effect of accuracy was significant such that responses were slower for incorrect responses, *F*(1,78) = 36.52, *p* < 0.001, η_p_^2^ = 0.319, BF_10_ > 100. The condition × interval interaction was also significant, F(1,78) = 6.06, *p* = 0.016, η_p_^2^ = 0.072, BF_10_ = 9.59, with pairwise comparisons indicating that the difference in response times between the CR and Control conditions was more pronounced in the no wait group (t = 7.48, *p* < 0.001, BF_10_ > 100) compared to the wait group (t = 3.39, *p* = 0.004, BF_10_ = 11.36), see Fig. 4. None of the other higher order interactions were significant (all *p* < 0.10).

Decision accuracy in the CCJ condition (M = 72.8%, SD = 12) was significantly worse than accuracy in the Control condition (M = 76.5%, SD = 9.9, *F*(1,77) = 25.16, *p* < 0.001, η_p_^2^ = 0.246, BF_10_ > 100. Accuracy for the no wait group (M = 72%, SD = 11.14) did not differ significantly compared to accuracy on the wait group (M = 76.92%, SD = 10.65), F(1,77) = 3.89, *p* = 0.052, η_p_^2^ = 0.048, BF_10_ = 1.45). Crucially, there was a significant condition × wait-interval interaction, F(1,77) = 13.35, *p* < 0.001, η_p_^2^ = 0.148, BF_10_ = 52.71, such that comparisons with Holm correction indicated confidence ratings only impaired participants in the no-wait group (t = 6.09, *p* < 0.001, BF_10_ > 100), but not the wait-interval group (t = 0.969, *p* > 0.99, BF_10_ = 0.27).

These findings replicate that of the previous experiments, in that concurrent confidence ratings slow responding and impair accuracy when there is no wait-interval between stimulus offset and presentation of the confidence rating. Importantly, by allowing participants time after stimulus offset to make their decision before presenting the response options, negative reactivity on decision accuracy is eliminated and only relatively weak effects are observed on response time. These findings suggest that rating one’s confidence at the same time as making a perceptual decision creates negative reactivity, rather than the complexity of the available response options.

## General discussion

Confidence ratings are one of the most frequently used measures of metacognition. Researchers have typically elicited such ratings assuming that they will not affect the underlying decision-making process. Underlying this assumption is the belief that individuals naturally monitor their confidence and reporting this confidence does not represent an additional computational burden. Recent evidence with RCJs has consistently shown that eliciting such judgments slows decision-making^9–14,23^, suggesting, at the very least, that when confidence ratings are elicited additional processing during decision-making occurs. Evidence that eliciting RCJs impacts decision accuracy is more equivocal. However, the effects of eliciting RCJs are likely to be muted by the fact that they occur after the decision and thus their impact is limited to carry-over effects across trials.

These experiments were the first to examine reactivity in confidence judgments made concurrently with the decision itself. Across three experiments, we showed a robust negative effect of CCJs on decision accuracy. In Experiment 2 we showed that reactivity to CCJs occurs in experimental contexts where reactivity to RCJs is not observed. Finally, in Experiment 3, we only observed reactivity to CCJs in terms of decision accuracy only when the primary decision and confidence judgment occur at the same time. Furthermore, we observed stronger evidence of response time reactivity when the primary decision and confidence judgment occur at the same time and only weak evidence when a delay was introduced. This finding suggests that presenting a confidence rating during the decision process interferes with decision-making.

### Reactivity

Reactivity is a significant problem facing the field of metacognition. There is evidence that all the most frequently used measures of metacognition are reactive, including confidence ratings^7–9,11^, judgments of learning^17–20^, and think-aloud protocols^30,31^. Confidence judgments elicited retrospectively have typically been shown to affect accuracy in complex problem-solving tasks (e.g., Raven’s Progressive Matrices)^7–9^, while slowing responding in simple perceptual discrimination tasks but having either a very small or no effect on accuracy^10,11,13,23^. However, we found no evidence that RCJs affected either decision time or accuracy in the current study. We may have failed to observe reactivity to RCJs for several reasons including the within-subject nature of our design or the fact that decision time was not speeded, only stimulus presentation time was speeded. Regardless, the effect of RCJs on decision accuracy appears to be smaller and less reliable in simple perceptual tasks compared to complex reasoning tasks. There are many reasons that one could hypothesize to account for this difference, including differences between speeded and slow complex tasks in terms of when confidence is calculated^23^, differences in the cues used in simple speeded tasks compared to slow and complex tasks^32^, and the strength of pre-existing beliefs concerning complex tasks compared to simple speeded tasks (i.e. most people have a sense of how good they are at puzzles, but not at discriminating between dots on a computer screen).

While we found no evidence of reactivity to RCJs, the current experiments provide robust evidence that confidence ratings elicited contemporaneously with the decision affect both accuracy and decision time. CCJs have been used by many researchers and are typical paradigms in both experimental psychology and neuroscience^26–29^. Reactivity effects are particularly concerning because they are unlikely to describe a simple shift in the distribution of accuracy, instead, some individuals are likely to be affected while some are not^7^. For instance, participants who base their confidence judgments on evidence that overlaps with the primary decision are less likely to have impaired accuracy when making confidence judgments. Indeed, several studies have shown individual differences in reactivity, specifically that individuals low in pre-existing self-efficacy are more susceptible to negative reactivity^7–9^. As a result, eliciting confidence judgments may change the underlying rank order of participants and could accentuate the effect of some experimental manipulations^33,34^ or individual differences characteristics.

### Mechanism of reactivity

Why does judging one’s confidence at the same time as making a perceptual decision result in less accurate decisions? Theoretical frameworks concerned with the accuracy of metacognitive judgments have shown that the alignment between confidence and decision accuracy depends on the cues (or evidence) used to judge one’s subjective performance and how diagnostic these are of actual performance^35^. It is reasonably well accepted within the metacognition literature that confidence judgments draw on a wide range of cues (i.e., evidence), many of which are unlikely to be used as evidence for the primary decision. For example, when making a confidence judgment people consider their global beliefs about their capabilities as well as their cumulative performance on the task thus far^3,35,36^. Both confidence judgments and decisions are affected by multiple features of the stimulus^36^, and people are able to strategically integrate these sources of evidence^37,38^. However, a wide range of evidence suggests that confidence judgments integrate evidence from multiple sources, including one’s experience with similar decisions in the past^36,39^ and global beliefs about one’s competence^7,38,40^. In addition, confidence judgments are independently affected by both decision accuracy and the difficulty of the task, with increasing confidence for correct decisions and decreasing confidence for incorrect decisions as the task becomes easier^41–43^. Thus, confidence ratings draw on a wide range of evidence sources, whereas the primary decision is based on the (relatively constrained) properties of the stimulus; this decoupling suggests that the rating one’s confidence directs attention to additional information, which does not facilitate, and indeed may impair, the primary decision-making process.

### Limitations

While the current findings show a clear pattern of negative reactivity to CCJs, we can only infer that this negative reactivity is driven by differences in the evidence used to make confidence judgments and the primary perceptual decision. Other mechanisms are, of course possible, for example it may be that, rather than considering qualitatively distinct evidence for the primary decision and the confidence judgment, the weight given to different aspects of the same evidence differs when confidence judgments are elicited. For instance, it may be that confidence judgments cause participants to give more weight to unhelpful evidence (such as those that cause so-called metacognitive illusions) than they otherwise would^44^. Future research may explore these possibilities by examining how reactivity is affected by the presence of features known to produce metacognitive illusions (e.g., font size^45^. Finally, while the current study provides some evidence of negative reactivity in a perceptual discrimination task, further work is needed to establish whether these findings generalize to different samples and task parameters. Indeed, the equivocal nature of reactivity findings thus far suggest that there may be important moderators and boundary conditions to consider.

## Conclusion

The current studies are the first investigation of reactivity to contemporaneously elicited confidence judgments. Across all three experiments, we found a clear pattern of negative reactivity, suggesting that these judgments interfere with participants’ ability to make accurate decisions. These findings suggest that confidence judgments are reactive, and this may be due to differences in the evidence used to make perceptual decisions and confidence judgments. This has both practical implications, in that the use of confidence judgments by researchers may be problematic in some circumstances, and theoretical implications, in so far as these findings imply that models of metacognition cannot view confidence judgments as a simple read out of the quality of the evidence used to make the primary decision.

## Data Availability

Data and materials are available on the Open Science Framework (https://osf.io/3s98e). These experiments were not preregistered.
